# Non-cognitive Support for Postgraduate Studies: A Systematic Review

**DOI:** 10.3389/fpsyg.2021.773910

**Published:** 2022-01-21

**Authors:** Jose Frantz, Jill Cupido-Masters, Faranha Moosajee, Mario R. Smith

**Affiliations:** ^1^Research and Innovation, University of the Western Cape, Bellville, South Africa; ^2^Department of Psychology, University of the Western Cape, Bellville, South Africa; ^3^Division for Postgraduate Studies, University of the Western Cape, Bellville, South Africa

**Keywords:** non-cognitive skills, postgraduate students, retention, interventions, systematic review

## Abstract

Retention of postgraduate students is a complex problem at higher education institutions. To address this concern, various forms of academic support are offered by higher education institutions to nurture and develop the pipeline of postgraduate students. The support provided to postgraduate students tends to emphasize academic support at times at the expense of psychosocial or non-academic support. Non-cognitive skills were underscored as integral to determining academic and employment outcomes and thus, may need to be investigated more. This manuscript reports on an attempt to filter and consolidate the literature reporting on interventions for postgraduate students that include the development of non-cognitive skills. A systematic review was conducted, because it enabled rigorous and replicable process of consolidating literature. Covidence software was used as a digital platform for the systematic review. The review was conducted at four levels as per the PRISMA guideline namely, identification, screening, eligibility and final summation. The filtration process attempted to answer the following research questions: (1) How are non-cognitive factors or skills defined? (2) Which non-cognitive skills were included in support for postgraduate (Masters and Doctoral) students in the higher education setting?, and (3) How have non-cognitive skills been included in support interventions provided to retain postgraduate students? Descriptive and theory explicative metasynthesis was used for the summation and data extraction. The primary finding was that the term non-cognitive was not used explicitly in the included studies to describe skills or factors supporting student retention. The discourse centered around support and social support as non-academic factors and skills. This suggested that non-cognitive skills were constructed as co-curricular and not integrated into the postgraduate academic project or core learning outcomes. The findings highlighted the distinction between non-cognitive skills and factors and illustrated how skills and factors operate at different levels with different spheres of influence. The formats of support provide an intersectional space where skills and factors are combined.

## Introduction

Universities around the world are grappling with retention and throughput of postgraduate students (Zewotir et al., 2015). The ability of an institution to retain a student throughout the life cycle from admission to completion is referred to as retention (Seidman, 2005). Retention of postgraduate students is a complex process affecting students at higher education institutions (Letseka and Maile, 2008; Kritzinger and Loock, 2012). The Council on Higher Education (CHE) in South Africa identified that retention and throughput were global concerns that were attributed to a range of factors that can act as barriers or facilitators of success (Council on Higher Education (CHE), 2018). Attrition (drop out) is an international challenge with a multifaceted foundation (Van de Schoot et al., 2013). Student retention is a key performance indicator for higher education institutions (Crosling et al., 2009). When students drop out, the losses include reduced revenue, reputational harm, lower returns on investment, and reduced funder confidence (Cook and Rushton, 2010). Gittings et al. (2018) reported that attrition ultimately impacts the economy of the country negatively.

Higher education institutions must examine the contributing factors that impact the retention of postgraduate students (Aljohani, 2016). Abiddin and Ismail (2014) highlighted that the engagement and understanding of students’ experience and perceptions of their studies is essential if we are to develop a healthy student-oriented environment. Similarly, Crosling et al. (2009) emphasized that student retention is contingent on actively facilitating student engagement and understanding student needs. The insights gained from student engagement should be incorporated into targeted student support.

Various forms of academic support are offered at higher education institutions to nurture and develop postgraduate students, and the pipeline. Support typically includes transferable skills training and research methodology support that are presented in the form of workshops and seminars (Aithal and Kumar, 2016). Exposure to networking opportunities, fellowships, and mobility are also available to doctoral candidates (Douglas, 2020). This author also identified financial assistance, work-study programs, and psycho-social support through existing student counseling services as forms of support (Douglas, 2020).

The support provided to postgraduate students emphasizes academic support at the expense of psychosocial or non-academic support Megginson (2009) identified that retention of students is dependent on both academic or cognitive ability, and non-cognitive skills (socio-emotional skills). Non-cognitive skills cover a range of abilities such as conscientiousness, perseverance, and teamwork (Lupton, 1998; Moyano et al., 2020). Non-cognitive traits and behaviors support students to make a success of their studies (Khine and Areepattamannil, 2016). Megginson (2009) underscored non-cognitive skills as integral to determining academic and employment outcomes. These skills are critically important to student achievement at all levels.

The quality and appropriateness of support is integrally linked to the infrastructure and resources available at an institutional level, as well as the strategic positioning of postgraduate studies (Moyano et al., 2020). The development and implementation of contextually appropriate interventions for postgraduates are dependent on clear needs assessments, insight into the student experience, consolidation of resources, and leveraging of available resources (Douglas, 2020). The extraction of empirical evidence from good quality literature reporting on interventions that include non-cognitive factors/skills in addition to academic support is critical to establishing an empirical basis for further program development. There is a growing body of literature reporting on the importance of non-cognitive skills that requires filtration. This manuscript reports on an attempt to filter and consolidate the literature reporting on support for the retention of postgraduate students that include non-cognitive skills and factors.

### Literature Review

The problems of retention and attrition have, in part, been addressed by examining (1) the thesis endeavor itself, (2) the supervision process, (3) quality assurance, and (4) student support. Wright (2003) identified the lack of scholarly recognition for the psychological nature of postgraduate studies and research processes. Dickson et al. (2011) concluded that supervision and postgraduate studies are impacted by psychological constructs. Ladany et al. (2013) further confirmed that that the impact of psychological processes on research and supervision already has been the focus of research. Similarly, demographic variables were reported to impact postgraduate enrollment, throughput and process experiences. For example, gender (Grace and Gouthro, 2000; Grant, 2003; Lee, 2008), race (Schroeder et al., 2009; Maton et al., 2011), religion (Berkel et al., 2007), and sexual orientation (Messinger, 2007) were previously investigated.

Psychological constructs, demographic variables, and contextual factors (e.g., SES) were incorporated under the broad umbrella of non-cognitive skills and non-cognitive factors that impact retention. For example, Dickson et al. (2011) looked at how attachment style related to the Working Alliance between students and supervisors and resultant success. The ability to forge a productive working alliance was reported in the late 90s as a “soft skill” that promoted completion (Armstrong et al., 1997). Smith (2004) reported that the working alliance between students and research supervisors was a proxy for attachment style. In other words, the working alliance was a manifestation of the quality of relationships and inherent coping styles (i.e., attachment).

Nagaoka et al. (2013) unpacked these concepts further into a framework of non-cognitive skills and highlighted the importance of context. The authors underscored that the context of higher education institutions must be considered as a contributor to the academic success of students. Dembo and Selif (2013) highlighted that non-cognitive skills for university students includes a range of attitudes, behaviors and strategies that students need to possess in order to do well academically. In addition, higher education institutions must also consider the socio-cultural background of the student. Megginson (2009) identified three categories of constructs that comprise non-cognitive skills. The first category was personality factors that included emotional stability, conscientiousness, and personality traits such as extraversion. The second category was attitudinal factors that included constructs like self-efficacy and intrinsic motivation. The third category was referred to as quasi-cognitive factors. These skills are considered emotional in nature, but involve cognitive processes. For example, creativity and emotional intelligence both involve cognitive processes that are non-linear and subjective in nature.

There is no consensus in the literature on a definitive definition of non-cognitive skills or factors that impact postgraduate student retention and success (Khine, 2016). A range of constructs and terms are used interchangeably to refer to non-cognitive skills such as, non-academic skills, socio-emotional skills, psychological factors, social support and contextual factors (García, 2016). A criticism of the literature is the failure to provide theoretical and operational definitions for non-cognitive skills examined in the respective studies. Thus, there is a need to distil from good quality literature, the skills with definitions or descriptions of non-cognitive skills, and factors that impact student retention.

Mouton (2011) stated that the university processes related to higher degrees constitute the necessary conditions to facilitate successful completion of postgraduate studies. He argued further that university processes and institutional conditions for the provision of postgraduate studies and an enabling environment are classified as non-cognitive factors. These factors in turn, impact student retention and throughput. Thus, universities, alongside other research-led institutions or organizations, and quality assurance agencies must become intentional about expanding their systematic exploration of retention rates among postgraduate students to include the impact of non-cognitive skills (National Planning Commission, 2013). The recommended inclusion of non-cognitive skills is aligned with the finding from research on postgraduate provision and capacity building identifying that student retention was impacted by academic or cognitive ability, and non-cognitive skills (socio-emotional skills) of students (Fynn and Janse van Vuuren, 2017; Farruggia et al., 2018).

### Impact of Non-cognitive Skills

As mentioned before, non-cognitive skills were underscored as integral to determining academic and employment outcomes, and thus may need to be investigated further. For example, personality functioning and personality factors were identified as non-cognitive factors. Judge et al. (2002) explored how self-esteem, self-efficacy, neuroticism and locus of control may all be indicators of a single, higher order personality construct that impacts postgraduate success. Senekal and Smith (2015) reported that the interaction between self-esteem and the predisposition to make use of social support (network orientation) significantly predicted the perception of research as stressful. These authors also reported that the quality of the working alliance was a product of the interaction between self-esteem and network orientation. Thus, personality factors impact student performance in postgraduate studies and remain a focus of further research.

Social support was identified as impacting postgraduate retention positively, because it reduces isolation (Kiguwa and Langa, 2009). Isolation was identified as problematic for postgraduate students, especially for female students in the social sciences students (Grix, 2010). Grant (2003) underscored that the non-cognitive aspects of the induction of students into the broader academic community and a warm, professional supervisory relationship were key to success. Students require more than just a transference of academic knowledge and skills, but also need emotional support (Manathunga, 2005). Feeling supported and attended to by supervisors repeatedly come up in the literature as non-cognitive elements which are central to a positive experience of postgraduate studies and throughput (Flynn et al., 2011).

Jairam and Kahl (2012) reported that feelings of helplessness, anxiety and loss of self-esteem contribute negatively to attrition. Barry et al. (2018) indicated that the subjective experience of completing postgraduate studies include psychological distress and emotional challenges. These authors argued that doctoral studies were particularly stressful and that failure to mediate the perceived stress negatively impact attrition rates and completion times. Morrison-Saunders et al. (2010) stated that emotion was a natural part of doctoral studies. Students start out excited and anxious about the journey ahead, and the world of opportunities that await them (Christie et al., 2008). The initial emotions are however affected by various experiences on the academic journey. The success of completing a doctoral degree weighs heavily on the ability to navigate and merge emotion (Walker and Thomson, 2010; Can and Walker, 2014). Jairam and Kahl (2012) states that gaps in the literature include doctoral candidate retention through social support as a mediator of stress, the role of academic colleagues, the role of family and friends, emotional support, and professional support. Institutions place less focus on emotion as it could heighten the “concern for the therapeutic rather than the pedagogic” (Beard et al., 2007, p. 237). A better understanding of the emotions that students experience while studying can inform student support and improve progress with their studies (Morrison-Saunders et al., 2010).

Walker and Thomson (2010) identified the formation of a scholarly identity as an important factor impacting student retention. Smith and Chitanga (2017) argued that identity formation is a psychological process that includes the process of internalization and integration of attitudes and views acquired during supervision and the research process. Higher education has both a social and academic sphere, and it has been found that the social sphere can lead to individuals leaving the institution. As an institution, there are two forms of integration that need to be considered and these are normative and structural (Oksavik et al., 2021). Normative integration refers to the communality in the frame of reference where all individuals have similar values and goals toward a communal outcome. These forms of integration impact the way students view their individual participation on their academic journey. This relates to their academic achievements which impacts subsequent employability and career development. Therefore, integration and identity formation are especially important to be aware of in the provision of support to postgraduate students.

Sverdlik et al. (2018) conducted a systematic review and concluded that non-cognitive factors can be categorized into internal and external factors. These authors identified the following external factors as having an impact on the Ph.D. experience: supervision; role of the supervisor in the doctoral experience; importance of supervisory fit; personal or social lives; departmental structures and socialization; and financial opportunities. The following internal factors were highlighted as having an impact on the Ph.D. experience: motivation; writing skills, emotion regulation strategies; academic identity; self-worth and self-efficacy (Sverdlik et al., 2018). The results from the review illustrate that the doctoral degree is multifaceted, and that both external and internal factors impact on the achievement and well-being of the candidate. This review empirically established that there is a body of literature reporting on non-cognitive factors and their impact on the doctoral experience (Sverdlik et al., 2018). These authors reported that postgraduate students experience numerous stressors such as, balancing relationships, finances, transport, and health. These stressors underscore that postgraduate students occupy many roles in addition to that of a student. The competing demands that result and manifest in sources of stress require coping skills and internal resources that are non-cognitive in nature. Thus, academic success and postgraduate studies do not take place in a vacuum and a complex set of non-cognitive skills are required to master the resulting challenges.

Non-cognitive skills impact a student’s ability to think critically about information, manage their time, get along with their peers and instructors, persist through difficulties, and navigate the different requirements and challenges that they may face throughout their postgraduate experience. It becomes evident that non-cognitive and cognitive skills interact and are not binary in nature which makes understanding its impact more difficult. Studies show a link between non-cognitive attributes and positive student outcomes in higher education. Megginson (2009) argued that an increased understanding and systematic exploration of the impact of non-cognitive factors related to student success will improve academic processes and support offered to postgraduates.

### Interventions

As mentioned before, research highlighted that students require more than only academic support, which raises questions about the responsibility of the postgraduate program and the institution to provide holistic support. Various forms of academic support are offered at higher education institutions to nurture the developing postgraduate student. The quality and appropriateness of support is integrally linked to the infrastructure and resources available at an institutional level, as well as the strategic positioning of postgraduate studies.

The development and implementation of contextually appropriate interventions for doctoral students are dependent on clear needs assessments, insight into the student experience, consolidation of resources and leveraging of available resources, as well as empirical evidence from good quality literature reporting on interventions that include non-cognitive factors/skills in addition to academic support. The development of non-cognitive skills is complex, and studies examining their development must account for this complexity. Merino et al. (2019) concluded that support interventions focusing on the development of non-cognitive skills should allow students time to practice and apply the knowledge. What becomes evident is that there is a need to assess the format of support interventions for postgraduate students and how non-cognitive factors and skills are incorporated into support programs.

## Method

A systematic review was conducted, because of the rigorous and replicable process for consolidating literature (Armstrong et al., 2011). Systematic review allowed for transparent and systematic methods to search for, identify and select relevant literature, assess it for methodological rigor and to summarize and analyze the data found in the research available (Moher et al., 2009). The preferred reporting items for systematic reviews and meta-analysis (PRISMA was used to strengthen the reporting on the review, Moher et al., 2009). The review was conducted at four levels namely, identification, screening, eligibility and final summation. Covidence systematic review software was used to capture data. Two independent reviewers conducted the review and two independent collaborators audited the review to ensure methodological rigor and coherence as recommended by Staples and Niazi (2007). Armstrong et al. (2011) underscored that multiple reviewers and external auditing can dispel concerns like selection and publication bias as well as, biased evaluation of studies for inclusion. Data was extracted based on the research questions.

### Review Question

The review was conducted in order to answer the questions:

(1) How have non-cognitive factors and skills been defined in postgraduate student retention?
(2) What non-cognitive skills were included in support for postgraduate (Masters and Doctoral) students in the higher education setting?
(3) How have non-cognitive skills been included in support interventions provided to retain postgraduate students?

### Inclusion and Exclusion Criteria

Studies reporting on non-cognitive skills included in postgraduate support were eligible. The studies had to include non-cognitive factors or non-cognitive skills as part of a support model or intervention for the retention of postgraduate students. Studies published between 2010 and June 2021 with postgraduate students (Master’s and Doctoral) as the target group were included in the review. The period of review is based on recency and the exponential growth of literature post 2010. Only primary studies were considered while reviews were excluded. A specific type of methodology was not used as an inclusion criterion. Studies had to be peer reviewed and published in scientific journals. Theses, conference proceedings and reports were excluded. Full text articles published in English and Afrikaans were considered based on the linguistic abilities of the review team. However, there were no articles published in Afrikaans identified on the relevant topic and inclusion criteria.

### Search Strategy

The following search strategy was employed to identify literature answer the review question:

(a) ***Keywords.*** In field of Title: “Postgraduate student” OR “Doctoral student” OR “Master* student” OR “Ph.d. student” In ANY FIELD: AND Attrition OR retention OR throughput In ANY FIELD: AND “graduate attributes” OR intervention OR strateg* OR “capacity building” OR “support model” OR “non-cognitive skill” OR “non-academic support” OR “academic support” OR resource OR “theoretical concept*” OR “postgraduate research training” OR “supervision”
(b) ***Databases***: The UWC library offers an integrated search facility, Ukwazi, that was used for the comprehensive database search across all databases to which the university subscribed. The following databases yielded results EBSCOhost EJS, Taylor & Francis Online, Taylor & Francis: Master, Academic Search Complete, DOAJ Directory of Open Access Journals, Elektronische Zeitschriftenbibliotek Frei zugangliche E-Journals, Taylor and Francis: Social science and humanities, SpringerLink Contemporary, Springer online journals, JSTOR Archive collection, Free medical journals, Springer link journals, Springer 2012 corporate journal collection, Pubmed Central, Springerlink contemporary – humanities, social, science and law, SpringerLink King Size, Springer for R&D Journals, Wiley online library and Free accessible social science journals.

### Review Procedure

The data base search identified titles that reflected the search terms. Duplicates were removed and titles were screened for relevance to the study. Titles that appeared relevant were retained and those that did not were excluded from further review. The abstracts of retained titles were screened according to the inclusion and exclusion criteria. Studies that satisfied inclusion criteria were retained and those that met the exclusion criteria were excluded. The full texts of included abstracts were retrieved and appraised for methodological rigor and coherence using Form D of the SFS scoring system (Smith, 2014). This Form D of the critical appraisal tool was developed to evaluate studies of different methodologies along a generic set of criteria. The composite score obtained by each study was expressed as a percentage and assigned a quality descriptor. For example, weak (0–40%), moderate (41–60%), strong (61–80%), or excellent (81–100%). The threshold for inclusion was set at 60%. All articles that met the threshold for inclusion were included for summation in the review.

### Data Analysis

A descriptive and theory explicative meta-synthesis was used as a method of summation. The operational steps in the meta-synthesis were adopted from Noblit and Hare (1988). First, the recurring themes and ideas were identified after reading the articles selected for inclusion in the study. Second, categories were created to organize the extracted data. Meta-synthesis is a rigorous method that allows for the aggregation and qualitative interpretation of the findings in the included articles (Walsh and Downe, 2005; Lachal et al., 2017). A meta-synthesis relies on a subjective interpretation of the findings (Erwin et al., 2011). Methodological rigor was maintained during the meta-synthesis through an impartial assessment of the findings by an independent reviewer, collaborative discussions and an understanding of the data.

## Results

### Process Results

**FIGURE 1:**
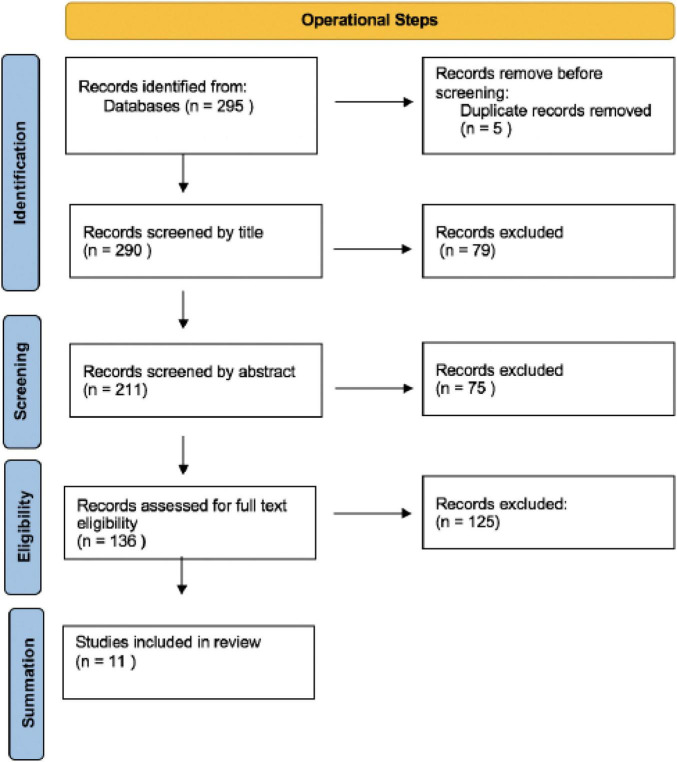
Adapted PRISMA flow chart (Adapted from Moher et al., 2009). The included studies are listed in Table 1 with the scores they were assigned in ascending order.

The search identified a total of 295 titles. These articles were exported to Covidence and independently reviewed by two reviewers. Conflicts that arose were discussed and a resolution was achieved through consultation between the two independent reviewers. Five duplicates were identified by Covidence and automatically removed. The remaining 290 titles were reviewed of which 79 were excluded, these titles did not meet the inclusion criteria. The abstracts of the remaining 211 records were screened based on the inclusion and exclusion criteria resulting in the exclusion of a further 75 records. A total of 136 full texts were retrieved and assessed for methodological rigor and coherence. Eleven articles met the threshold for were inclusion in the final summation and the remaining 125 were excluded. All the articles included were above the threshold of 60% and achieved quality descriptions of ‘strong’ (61–80%) or ‘excellent’ (80% and higher). The process results are graphically illustrated in Figure 1.

**TABLE 1:** Methodological quality.

No	Citation	Score %	Quality
1	Caliskan and Holley, 2017	64	Strong
2	Cisco, 2020b	69	Strong
3	Hoffman and Julie, 2012	69	Strong
4	Holley and Caldwell, 2012	64	Strong
5	Le Roux, 2018	67	Strong
6	McAlpine and Asghar, 2010	71	Strong
7	Menzies et al., 2015	91	Excellent
8	Mullen et al., 2010	78	Strong
9	Vacek et al., 2021	73	Strong
10	Viđak et al., 2017	73	Strong
11	Williams et al., 2017	89	Excellent

### Meta-Synthesis

#### Definitions of Non-cognitive Skills or Factors

The primary finding was that the term non-cognitive was not used explicitly in the included studies to describe skills or factors supporting student retention. The discourse centered around support and social support as non-academic factors and skills. This suggested that non-cognitive skills were not integrated into the postgraduate academic project or core learning outcomes. This is most evident in the common use of the term non-academic as a synonym for non-cognitive. Support or transferable skills training involving non-cognitive skills and factors were co-curricular and therefore not given the attention it deserved.

A further observation was that there was a distinction between skills and factors as the direct subject with non-cognitive being a qualifier or an adjective. There was no consensus on the definition of the adjectival use of non-cognitive. This impacts the ability to compare the literature as different authors and sources use similar terms whilst referring to very different denotations or used different terms referring to similar denotations. In addition, there was a range of connotations associated with the varied terms that were used contributing to the lack of clarity. The terms were not always defined consistently and authors assumed that their readership would have a clear reference for the qualifier non-cognitive. What emerged clearly was that the definition was based on what it is not, i.e., it was not cognitive in nature. To some extent there was an attempt to acknowledge the interplay between cognitive and non-cognitive resulting in the use of the term quasi-cognitive.

The most striking finding was that there was a patterned use of the terms, skills and factors. This distinction albeit implicit, was very useful as it spoke to the direct subject and contributed to a more usable differentiation of the collective term used for non-cognitive skills and factors. The differentiation between skills and factors focus on the planes in which they operate or their respective spheres of influence. Skills refer to capacities and abilities that the individual student possesses. These skills can be either intrapersonal or interpersonal. Factors refer to aspects that influence the postgraduate student and are located in the contextual reality of the students such as programmatic factors or institutional factors or extra-institutional factors. Programmatic factors relate to structural and operational aspects of the program that impact student retention, e.g., governance and supervision. Institutional factors relate to aspects of the institution that impacts retention, e.g., institutional culture, funding and quality assurance. Extra-institutional factors relate to the broader socio-political context and factors that would influence the higher education sector at large, the employment market, access to and participation in postgraduate studies, as well as historical influences such as the vestiges of Apartheid in South Africa that result in unequal capacities and systematic historical disadvantage or advantage. Factors and skills work in a reciprocal manner and a complex interplay result that may be distinctive to an individual student, a program, an institution or the section nationally and globally. Thus, the format of support becomes crucial it includes both the development of skills and the leveraging of factors that provides an intersectional space. Within the format or intervention space the skills (intrapersonal and interpersonal) interact with contextual factors (programmatic, institutional, and extra-institutional) to promote retention and throughput.

#### Non-cognitive Skills Included in Postgraduate Support

Twelve non-cognitive skills were identified at the level of the student. Table 2 summarizes the non-cognitive skills that were identified from the included articles. The table also separates the skills into intra- and interpersonal, and indicates the source documents where the particular skill was mentioned. The skills are further introduced in order of descending frequency of mentions in source documents.

**TABLE 2:** Non-cognitive skills at a student level.

Student factors	Source	Description
**Intrapersonal**		
Intrinsic motivation	1, 3, 4, 7	The capacity to draw on internal resources to remain motivated in the pursuit of postgraduate studies. Implies an internal locus of control.
Self-efficacy	8, 10, 11	Self-efficacy is directly related to how students perform and achieve confidence in their ability to complete postgraduate studies. It includes the belief that one has the power to effect change, have mastery and agency to complete postgraduate studies. Correlated with self-esteem.
Identity construction	7, 8, 11	The process of integrating values, experiences and attitudes to form a clearly defined sense of self in relation to the postgraduate experience.
Sense of belonging	3, 9	Positive identification with the institution, program and course of study.
Self-esteem	11	Related to personal beliefs about skills, abilities, social relationships, and future outcomes Includes self-confidence.
Self-monitoring	7	The capacity to track progress and subjective experiences in the context of postgraduate studies.
Self-actualization	3	The ability and drive to pursue personal goals.
Values	11	The values and principles students considered as important. This impacts appraisal of perceived benefits and sense making of experiences.
**Interpersonal**		
Ability to establish a working alliance	1, 2, 3, 4, 5, 7, 8	The skills to establish and maintain a productive relationship with supervisors.
Networking skills	1, 4, 11	Skills set to socialize and engage others toward mutual goals.
Relational capacity	5, 7, 9	The ability to establish and maintain supportive relationships with family and friends.
Network orientation	3, 11	Predisposition to make use of support.

Seven non-cognitive factors were identified at the level of academic program and institution. Table 3 summarizes the non-cognitive factors with a description and the source articles. As before source documents are indicated and frequency of mentions determine the order of presentation. These factors may be operant at both a programmatic and institutional level. The location is determined by where the authors placed the factor.

**TABLE 3:** Non-cognitive factors at a programmatic and institutional level.

Institutional factors	Source	Description
**Programmatic**		
Professional guidance	1, 2, 4, 7, 11	Exposure to the professional world of work and the creation of opportunities to develop market related skills.
Supervisory practice	1, 2	Positive experiences of supervisory practices.
Staff relationships	4	Positive and collegial relationships with staff in the postgraduate program.
**Institutional**		
Research culture	1, 2, 3 4, 6	The values subscribed to in the program and institution in relation to research integrity and research productiveness.
Information sharing	6, 7, 8, 11	Clear and transparent communication about important aspects that impact the postgraduate student.
Institutional identity	2, 6, 10	A coherent and positive identity as an institution, faculty or study program.
Financial support	1, 6, 7, 10	Assistance with study finances and assistive study devices through funding instruments.

Six forms or formats of support to postgraduate students were identified. Table 4 identifies the formats of support or interventions where non-cognitive skills have been incorporated. Format is presented in descending order of frequency of mentions.

**TABLE 4:** Formats of support.

Format	Source	Description
Mentoring/coaching	1, 2, 4, 5, 7, 8, 10, 11	Formalized programs to provide guidance offered by staff.
Communities of practice	1, 4, 6, 8, 9, 11	Establishing a community or network that aims to reduce isolation, promote collaboration and knowledge exchange.
Skills training	2, 4, 6, 8, 11	Transferable skills training on a range of topics.
Student support services	1, 2, 3, 7, 10	Formalized services for specific student needs, e.g., psychotherapy, financial aid, health etc.
Program activities	4, 6, 7, 10	Activities arranged within the ambit of the academic program, e.g., case conferences, symposia etc.
Peer mentoring	4, 8, 7,	Formalized opportunities to consult and advise fellow students.

## Discussion

The aim of this systematic review was to identify the non-cognitive factors that have been included in the support interventions for postgraduate student retention. The study further aimed to understand how non-cognitive skills and factors were defined in the context of postgraduate studies.

### Definitions

There was no consensus on the definition of non-cognitive or non-academic skills and factors in student retention in the broader literature. This impacts the ability to compare the literature as different authors and sources may use similar terms whilst referring to very different denotations and connotations. This suggested that non-cognitive skills were limited to the co-curricular space and not integrated into the postgraduate academic project.

The findings made an important distinction by deconstructing the phrase, “non-cognitive skills or factors.” First, “non-cognitive” is an adjective that qualifies a set of skills or factors. The term denotes that this set of skills and factors are not primarily cognitive in nature. In essence, the qualifier, non-cognitive, is used to refer to skills and factors impacting student retention. The term, non-cognitive, was not used explicitly in the included studies. This was different from the broader body of literature where the term was used explicitly and frequently (e.g., Khine and Areepattamannil, 2016).

The data extracted from included studies identified two other collective nouns used as a qualifier for factors and skills namely, (a) non-academic and (b) support including social support. The binary distinction between academic (cognitive) and non-academic (non-cognitive) is problematic in that it creates the impression that there is no interaction between these skills. An outflow of this is that skills or factors that are less easily placed into one of these discrete categories are less frequently included in research. This results in a biased enquiry and an inability to develop a holistic and comprehensive understanding of factors that impact student retention. Moreover, the reciprocal nature and the intersectionality between cognitive and non-cognitive are under-appreciated in both theory and intervention.

The results indicated that the phrase, “skills and factors” has been used as an umbrella term or collective noun. The distinction between skills and factors were not always made explicitly resulting in the increased use of the phrase, “skills and factors.” More concerning the terms skills and factors were used interchangeable which negated the unique placement and spheres of influence that each of these concepts hold.

A pattern was observed in the use of the phrase as opposed to the use of the individual terms. On the one hand, studies that examined both skills and factors tended to use the phrase to for ease of reference. At a conceptual level, these studies did not make the distinction between skills and factors and were measuring them cross-sectionally and examining them in correlational terms. Review studies that attempted to filter this body of literature tended to use the phrase to organize the extracted data as a coherent whole without unpacking the collective term and the implications thereof for further conceptualization and research. In part, this can be attributed to the use of descriptive meta-synthesis instead of theory-explicative or theory-building meta-synthesis as recommended by Sandelowski et al. (1997). This was a major limitation of review studies as the secondary nature of such studies was well-placed to help make these distinctions clearer. In turn, such findings would shape further research. On the other hand, studies that examined specific skills or factors and their relative impact on student retention, tended to name the specific skill or factor.

### Categorization

The findings identified 19 non-cognitive skills or factors that support retention of postgraduate students. The skills or factors were categorized based on the different planes in which they operate. Two primary ways of categorization were identified namely (a) internal versus external, and (b) student, programmatic and institutional levels. These categorizations were applied to both skills and factors without making a further distinction between skills and factors. There were significant overlaps in these two ways of categorization that resonated with the categorizations proffered by Megginson (2009) and Sverdlik et al. (2018).

The student level refers to an intrapersonal and interpersonal level. The first two categories proposed by Megginson (2009) include personality factors and attitudinal factors that both refer to factors operant at the student level. The skills in these categories are thought to be emotional in nature and are considered non-linear and subjective. Thus, they are seen as intrapersonal or located within the personality and psychological make-up of the student. The personality also manifests in the interpersonal domain. Student level factors are similar to the internal factors articulated by Sverdlik et al. (2018).

The programmatic level refers to the context within the academic program whilst the institutional level refers to the broader university which includes the administration, faculty and institutional offices. At a programmatic level, supervision is an important strategy to develop capacity in postgraduate students through supervised research. At an institutional level supervision is an undertaking that is governed by the postgraduate policy of the institution and the administrative procedures and processes to guide the provision of an enabling environment for postgraduate research and study. External factors proposed by Sverdlik et al. (2018) are similar to the programmatic and institutional level factors. The external factors identified by these authors related to supervision and departmental structures. Supervision is both a programmatic issue and an institutional issue. Sverdlik et al. (2018) also included the personal and social lives, as well as socialization and financial opportunities. These factors are external to the student and related to a broader context than the institution. Le Roux (2018) argued that it was helpful for students to distinguish between personal, social and institutional levels. This will enable them to identify expectations within each of these categories and draw on skills and factors that operate within these categories. Postgraduate students will be able to prioritize and balance seemingly competing demands emanating from these levels. Doing so, enables them to be better able to accomplish their goals and experience less stress. In short, the use of categories is useful when examining factors and skills that impact student retention.

### Non-cognitive Skills

Eight intra -personal skills were identified while four interpersonal skills were identified.

### Intrapersonal Skills

Intrinsic motivation, identified in articles 1, 3, 4, and 7, refers to the capacity to draw on internal resources to remain motivated in the pursuit of postgraduate studies. From the source articles intrinsic motivation was described as an internal determination to complete a postgraduate degree. This resonates with the broader literature where a clear relationship was postulated between a student’s intrinsic motivation and the likelihood of successful completion. Intrinsic motivation is a complex construct that incorporates many aspects such as an internal locus of control. Intrinsic motivation was referred to as the combination of several factors such as self-confidence and belief in oneself (Hoffman and Julie, 2012). Students experience different levels of internal motivation at different times within their postgraduate journey which contributes to decision making about continuing or persevering when things become challenging or to end their studies (Christie et al., 2008).

Self-efficacy, self-esteem, and self-monitoring were a cluster of closely related and somewhat overlapping skills identified in the summation. Self-efficacy, identified in three articles, is directly related to how students perform and achieve confidence in their ability to complete postgraduate studies. It includes the belief that one has the power or capacity to effect change that would promote the successful completion of postgraduate studies. Self-efficacy was closely related to the Agency refers to the capacity to influence your environment and mastery refers to the acquisition of skills required in the course of studying. Self-efficacy was significantly correlated with self-esteem and self-confidence in the broader literature on student retention (Senekal and Smith, 2015). Self-esteem, identified in article 11, was related to personal beliefs about skills, abilities, social relationships, and future outcomes. Self-monitoring, identified in article 7, refers to the capacity to track progress and subjective experiences in the context of postgraduate studies. The capacity to monitor oneself in such a manner is based on awareness, insight and planning ability. This cluster of skills resonates with studies focusing on non-cognitive factors (Khine, 2016). Moyano et al. (2020) argued that non-cognitive skills cover a range of related abilities such as conscientiousness, and perseverance. This cluster of skills are thought to be outcomes or manifestations of self-actualization. Self-actualization was identified in article 3 and related to the ability and drive to pursue personal goals. This psychological construct refers to an inherent drive which is central to specific humanistic theories of personality. The literature does not engage with the different levels at which the identified non-cognitive skills are presented, e.g., skills versus structural personality constructs. Similarly, the lack of engagement with the nature of constructs, as complex versus simple constructs, impacts conceptualization and instrumentation that in impact the quality of the data and findings. The article also did not engage with the classification of the constructs and the extent to which these constructs can be understood to have singular or multiple meanings and whether it can be rated in a singular or in multiple ways. The lack of conceptual validation of these constructs detract from the resulting measurement and analyses.

Identity construction was identified in three articles. Distilling a defined identity as a postgraduate student and as a researcher is a process. This process entails the integration of values, experiences and attitudes to form a clearly defined sense of self in relation to the postgraduate experience and the likelihood of succeeding (Oksavik et al., 2021). The literature reporting on postgraduate retention identified that the transition to postgraduate studies necessitates the development of a new identity as a researcher and postgraduate student (Walker and Thomson, 2010; Smith and Chitanga, 2017). This process is also complex and challenging at a subjective level. McAlpine and Asghar (2010) underscored that students expressed uncertainty in their new role as postgraduates and that they find it challenging to formulate an academic identity while balancing work, studies and life.

Networking skills, identified in articles 1, 4, and 11, entail a set of abilities that enables the student to form social and relational connections with others toward mutual goals. Senekal and Smith (2015) argued that networking skills were necessary to establish connections, but not sufficient. These authors argued that network orientation was a key ingredient. Network orientation is the predisposition or proclivity to make use of networks and support. This becomes a prerequisite for the use of skills. Network orientation was described and identified in articles 3 and 11, but not explicitly named as such. In addition to network orientation the authors in article 11 identified that the value system of the postgraduate students was important to consider. The values and principles students subscribed to impacts how they perceive network and support opportunities as beneficial and meaningful. These perceptions are value-based and determine their subsequent behavior and engagement.

Sense of belonging was identified in two articles. Positive identification with the institution, program and course of study fostered a sense of belonging that in turn promoted success. This resonated with Mullen et al. (2010) who reported that student confidence peaked when a sense of belonging was evident. A sense of belonging positively impacted on overall success.

### Interpersonal Skills

The ability to establish a working alliance was identified in seven articles (1, 2, 3, 4, 5, 7, and 8). The authors referred to a set of skills that enables the student to establish and maintain a productive relationship with supervisors and staff (faculty). This set of complex skills resonates with the literature reporting that supervision is a relationship and that students who possess good interpersonal skills and relational capacity are more likely to navigate the supervisory relationship and ultimately succeed (Dickson et al., 2011). Armstrong et al. (1997) identified working alliance as a soft skill that was integral to success. Smith (2004) further demonstrated that working alliance was a proxy for attachment style, i.e., the capacity to establish relationships and the quality of those relationships.

Relational capacity was identified in articles 5, 7, and 9. The focus was extended to the family and friends. The ability to establish and maintain supportive relationships was an important student factor that was closely related to their socialization and personal family and social background. The skills and capacity of the student to form relationships must not be interpreted without considering the nature of their social and familial contexts. This finding illustrates how internal student factors interact with external factors (Sverdlik et al., 2018). Silinda (2018) identified students’ ability to complete studies were enhanced when support was provided by family, peers, supervisors, and the institution. Productive relationships provide students with the ability to solve challenges, access relevant knowledge, and concentrate on the completion of their thesis.

The identified skills were at times a singular, discernible skills and at other times a set of skills. Thus, there is a hierarchy of intra and interpersonal skills that requires a level of abstraction and a good working knowledge of personality theory and the canon in psychology. Thus, when examining or attempting to understand the impact of individual factors, it must be conceded that these factors are psychological in nature. Further intervention and research must engage with the theoretical and operational definitions of these constructs in order to achieve higher levels of methodological rigor and coherence, as well as more greater relevance of the findings.

### Programmatic or Institutional Factors

The findings indicated seven non-cognitive factors that were operant at a programmatic or institutional level. It is understood that these factors operate at both levels, but they were presented here in terms of where the included articles located their primary impact. Three factors were located at a programmatic level and four at an institutional level.

Supervisory practice was identified in two articles. Positive experiences of supervision were identified as a factor impacting success and retention. The provision of good quality supervision is operant at a program or institutional level. The extent to which supervision is interrogated and quality assured falls within the ambit of the institution at large even though individual supervisors operationalize the supervisory relationship with the student. This finding resonated with McAlpine and Asghar (2010) who argued that a student’s positive relationship with the supervisor had a direct influence on whether a student decided to complete their degree (retention). These authors argued that students’ good supervisory practice clarifies expectations and shares other institutional information that in turn enables the student to feel comfortable and secure with their supervisor and at the institution. Similarly, Cisco (2020a) reported a need for a positive and supportive supervisor relationship. The impact of good quality supervision was extended to proposed ways in which supervisors could assist in overcoming imposter syndrome in postgraduate students. It is important to explore whether the programmatic and institutional aspects come through in the largely dyadic supervisory relationship.

Institutional identity was reported in three articles. It was argued that the nature of the institutional identity and the values subscribed to facilitated student retention. A coherent and positive institutional identity manifests at all levels of the university and sets the tone of the institutional culture. Similarly, the research culture of the institution was identified in five articles (1, 2, 3 4, and 6). The values subscribed to in the program and institution in relation to research integrity and research productiveness were identified to have an impact on student retention and performance. This finding resonates with literature reporting that institutional identity inclusive of the research culture at the institution, determined the nature and quality of support to postgraduate students that in turns impacts retention (Hoffman and Julie, 2012; Caliskan and Holley, 2017; Viđak et al., 2017). Menzies et al. (2015) argued that a positive institutional identity and research cultures fostered a positive relationship with institutional support programs that in turn allowed for ease of integration into postgraduate studies and promoted completion.

The nature and quality of the relationships staff establish with postgraduate students was identified as impacting retention. When staff relationships are positive and collegial in the postgraduate program, students felt respected and were more likely to make instrumental use of the staff as resources. However, when staff relationships were negative and unproductive, students reported struggling more at a personal and academic level. For example, Caliskan and Holley (2017) found that students reported negative experiences and negative impact on their performance when staff (faculty) lacked knowledge about the university and its policies or support systems in general. Thus, staff relationships are expected to be a source of information and support. The unmet expectation adversely impacts progress and student satisfaction with the experience of postgraduate studies. A criticism of the broader literature focused on faculty or academic staff members in advising or supervisory roles. The postgraduate student interacts with both research and instructional staff, as well as professional support staff. Thus, the impact of staff relationships must be examined systematically at all levels of the university organogram.

Information sharing was identified in article 6, 7, 8, and 11. A clear and transparent method of communication strategy promotes retention of postgraduates. Effective communication strategies should include information about important aspects that impact the postgraduate student. Such communications must also be delivered in methods, formats and on platforms that students are typically used by students. In other words, information sharing must be student-centered and contextually relevant. This resonates with the broader literature on effective communication and information sharing with student populations (Vacek et al., 2021; Williams et al., 2017). Caliskan and Holley (2017) went further to refer to information sharing as information support. Menzies et al. (2015) reported that students found information support to be integral to their success.

Professional guidance included advice on careers and exposure to the professional world of work. The creation of opportunities to develop market related skills was also identified as an important facet of professional guidance. Professional guidance was provided in the context of mentoring and role modeling. The institutional stance to guiding and orienting postgraduates to the world of work was identified as a factor that can impact retention and subsequent success. This finding resonates with literature where increasingly students report professional guidance to be an expectation albeit implicitly held (Williams et al., 2017).

Financial support refers to information about and material assistance with study finances and the procurement of assistive study devices. Practical support and information support was reported to positively impact on student’s ability to navigate funding instruments, acquire the technical skills to make applications for funding and the ability to secure grant awards. Financial support was not only limited to tuition, living and research expenses, but extended to include conference attendance, mobility experiences, as well as writing and editorial support. The global pandemic highlighted the need for tailored and extended financial support and access to financial support opportunities. This finding resonated with the literature reporting on the role of financial support in student retention. For example, Caliskan and Holley (2017) argued for a nuanced and comprehensive approach to financial support for study and living expenses as well as operational research expenses.

Skills and factors operate in different levels and have different loci of control given where they are situated. So, non-cog skills can be developed within a person whereas non-cog factors must be cultivated as part of the institutional culture or stance toward postgraduate education and research training. There is also an interaction and reciprocal influencing between factors and skills.

### Forms of Support

The findings identified six forms or formats of support or interventions where non-cognitive skills have been incorporated.

**Mentoring and coachin**g were the most frequently identified format for developing non-cognitive skills. Mentoring was described as a formalized support initiative in which staff form a relationship with postgraduate students. The mentoring relationship entails an intentional process of guiding and developing the mentee. Mentoring programs are an effective strategy to retain students (Menzies et al., 2015).

Mullen et al. (2010) identified mentoring as an integral component in support programs for doctoral students. Mentoring provided the potential for a long-lasting relationship to learn about discipline-specific information, life experiences and possible future careers. Mentoring was associated with increased confidence, more realistic expectations of the postgraduate program, overcoming imposter syndrome, increased sense of community and belonging consolidating academic identity, and the development of personal and professional skills needed for success (Viđak et al., 2017; Cisco, 2020b).

The impact of mentoring can be enhanced through matching mentors and mentees based on race, ethnicity, gender, and academic discipline (Holley and Caldwell, 2012). Mentors who volunteer were motivated by their own positive experiences of mentoring and were able to replicate the mentoring they received. Team-based mentoring was found to increase effectiveness of the program as participating students benefit from having a staff (faculty) mentor and a peer mentor (Caliskan and Holley, 2017). This provides a comprehensive foundation for development at various levels. As mentioned before, the student’s willingness to be a part of the program influences the success of mentoring initiatives. The lack of personality fit and challenging schedules limit the impact of mentoring.

Mentoring was also found useful to integrate international students provided that mentors are trained in cross-cultural aspects, had personal experience in being an international student themselves and have insight into university processes pertaining to the international students (Menzies et al., 2015). These authors argued that mentors can assist international students with various discipline specific needs and social support. Social support includes information support, assisting to find accommodation, friendship building, integration and reducing culture shock related to the country and institutional culture.

**Peer mentoring**, the least identified format, provides formalized opportunities to consult and advise fellow students. Peer mentorship is guided by four main principles: (1) assistance with academic transition – simplifying institutional expectations; (2) assisting students to learn independently and manage their time in a new environment; (3) introducing students to the support services that the institution has to offer and (4) providing a comfortable space where students can gain confident. Collaboration and dialogue are the main foundations of peer mentoring. The peer mentors benefit by gaining more opportunities to develop leadership skills, teamwork and interpersonal skills within a cross-cultural context. Beneficiaries of the mentoring program reported feeling secure and confident (Menzies et al., 2015). It was also recommended that this kind of intervention could be used with all students as it promotes feelings of belonging and security. Holley and Caldwell (2012) identified that the peer mentoring relationship was deemed most useful when a friendship formed as the support was at various levels. Peer mentoring reportedly strengthened communication, interdependence, critical thinking and self-management (Mullen et al., 2010). These authors argued that peer mentoring also assisted with how to provide and receive constructive feedback and mediate power hierarchies. The essence of the intervention is reciprocity.

**Communities of practice** was recommended in six articles (1, 4, 6, 8, 9, and 11) as it established networks. Such networks are effective in reducing isolation, as well as promoting collaboration and knowledge exchange. The studies included in the summation referred to the creation of a safe space in which students can excel and learn from one another. Through such non-cognitive support measures students feel that they form part of a broader community. The focus on communal activities provided students with the opportunity to network and meet other students and academics in various disciplines. These initiatives create an environment in which students experience the reported benefits of social support (Menzies et al., 2015). Students can share their experiences and the group can normalize those experiences and become a consultation group that assists in problem-solving and with testing out ideas. This knowledge sharing was deemed useful as there was a certain level of interdependence on one another to make a success of the postgraduate degree (Caliskan and Holley, 2017). Milestones and successes can be shared and celebrated. First-generation students especially benefit from communities of practice as they may not have individuals in their social and community networks who have a reference or shared experience to draw on (Rae and Smith, 2015). Students reported experiencing deeper levels of empathy and understanding in these groups when alumni were invited (Williams et al., 2017). McAlpine and Asghar (2010) reported that students became self-aware and started developing an academic identity through networking, social events and peer interaction. Tuasikal and Patria (2019) highlighted the need for interaction with peers that are completing similar work or find themselves in a similar position. In short, communal gathering and networks constitute communities of practice that have a positive influence on retention (Lovitts, 2005).

**Skills training**, identified in articles 2, 4, 6, 8, and 11, is offered to postgraduate students on a range of topics including research methodology, governance and academic skills (e.g., project management). Skills training is provided in workshop format. The workshop content focused largely on academic or methodological issues. The format, facilitation style and small group size contributed to the creation of a research culture in which students learnt how to give and receive feedback, talk about their work and practice academic debate and consultation. Transferable skills training can incorporate many non-cognitive factors and promote the development of non-cognitive skills (Vacek et al., 2021). The challenge is that facilitators and organizers must be intentional about leveraging transferable skills training as a platform for developing and supporting the student holistically and incorporating non-cognitive aspects.

**Program activities** (identified in articles 4, 6, 7, and 10) referred to specific actions taken by the staff (faculty) to promote retention. Examples included academic lectures, social gatherings, professional development events, case conferences, symposia and brown bag lunches (Caliskan and Holley, 2017; Viđak et al., 2017; Williams et al., 2017). Including these activities into academic programs conveyed to students that they were held in mind and accommodated in the program and by extension institution. These activities promoted the development of various non-cognitive skills and became a resource in and of itself. This in turn facilitated retention.

**Student support services** included formalized services for specific student needs, e.g., psychotherapy, financial aid, and health. Institutional support reportedly reduces stress which leads to greater success (Williams et al., 2017). Psychosocial wellness in education has become an important part of good quality education (NDP, 2030). Thus, student development and support services that promote and support psychosocial wellness are increasingly shown to improve retention (Nash and Sacre, 2009; Farrington et al., 2012). Psychosocial support can positively facilitate social and academic adjustment and transitioning in postgraduate students (Gray and Swinton, 2017). Conversely, a lack of support becomes a barrier to participation in and adjustment to postgraduate education. Support services must address cultural diversity, emotional intelligence, accessing and using social support, and emotional well-being in their programs as these have become critical in student retention (Williams et al., 2018).

The core finding was that the formats of support constituted a space where non-cognitive skills and factors intersected with one another, and with cognitive factors. Thus, attention and intentionality in planning formats of support and intervention is critical to the success as evidenced by retention and throughput. However, the studies neglected to address extra-institutional contexts which is key in the South African context.

## Conclusion

The review confirmed that there is a growing body of literature on non-cognitive skills and factors for student retention. The growth in this body of literature is encouraging and the findings underscore the need to transition from an exploratory mode to a more systematic exploration of how non-cognitive factors can be harnessed in holistic support programs or interventions.

The binary nature of the term non-cognitive and its synonyms (non-academic, social support) poses a challenge as it underplays the interaction between factors in the two categories. It also results in a biased enquiry into skills and factors that are more readily categorized into one of the domains.

The findings suggest a clearer demarcation between skills and factors. Skills were operant within the intrapersonal and interpersonal sphere of influence. In this category, personality constructs and specific skills were identified. Non-cognitive factors were operant at a programmatic and institutional level. The findings underscored the importance of factors in the context of the program and institution.

There was empirical support for the impact of non-cognitive skills and factors on student retention. Six formats of support were identified that were well established in the literature and supported for its reported impact on retention. These formats provided a platform for the development of skills and the leveraging of non-cognitive factors to create an enabling environment for postgraduate students.

Supervision was only identified as a programmatic or institutional factor. Supervision was constructed as a dyadic interaction between the supervisor and student for which they take personal responsibility with some degree of accountability at a programmatic level. Supervision was not seen as a relationship that could directly and intentionally effect change at a personal and professional level that in turn promotes retention. Thus, supervision must be examined further to fully appreciate the multifaceted nature thereof and to leverage the benefits that can be attained through an intentional use of supervision to promote the development of non-cognitive skills.

### Limitations

The present study excluded articles that required payment to view which might have introduced publication bias. The data extracted did not include statistical results or effect sizes. Given the variation in sample sizes, this was considered secondary to the aim of identifying components that could contribute to developing a conceptual understanding of the role of non-cognitive factors. The study did not include a theoretical framework as the intention was to distil components for subsequent theory building and developing conceptual frameworks. The study identified the lack of theoretical definitions in included articles which detracted from the statistics and meaningful comparisons thereof. Nevertheless, the lack of systematic engagement with the reported statistics was a limitation.

### Recommendations

The development of interventions or support programs for postgraduate students must adopt an empirical basis in which the conceptualization is characterized by a careful and intentional clarification of the skills and factors to be targeted and leveraged to achieve retention and throughput. Future research in the area of student retention should focus on the good conceptualization of non-cognitive skills and factors and how these in turn are integrated into comprehensive and holistic operational frameworks and programming for student retention.

## Data Availability Statement

The original contributions presented in the study are included in the article/supplementary material, further inquiries can be directed to the corresponding author/s.

## Author Contributions

JF contributed to the conceptualisation of the review as well as the coordination of the review, fieldwork, and data extraction, provided leadership and input to the review team at each stage of the project and the conceptualization of the manuscript, contributed to the write up and technical aspects of the article, and approved the submitted version. JC-M contributed to the conceptualisation of the review, fieldwork, data extraction, draft write up, revisions and editing of the manuscript, contributed to the write up and technical aspects of the article, and approved the submitted version. FM contributed to the refining of the conceptualization of the review when devising an operational plan for fieldwork, coordination of the fieldwork and data extraction, contributed to the write up and technical aspects of the article, and approved the submitted version. MS contributed to the conceptualization of the review, fieldwork, data extraction, draft write up, revisions, and editing of the manuscript, provided leadership and input to the review team at each stage of the project and the conceptualization of the manuscript, contributed to the write up and technical aspects of the article, and approved the submitted version. All the authors contributed to the article and approved the submitted version.

## Conflict of Interest

The authors declare that the research was conducted in the absence of any commercial or financial relationships that could be construed as a potential conflict of interest.

## Publisher’s Note

All claims expressed in this article are solely those of the authors and do not necessarily represent those of their affiliated organizations, or those of the publisher, the editors and the reviewers. Any product that may be evaluated in this article, or claim that may be made by its manufacturer, is not guaranteed or endorsed by the publisher.
